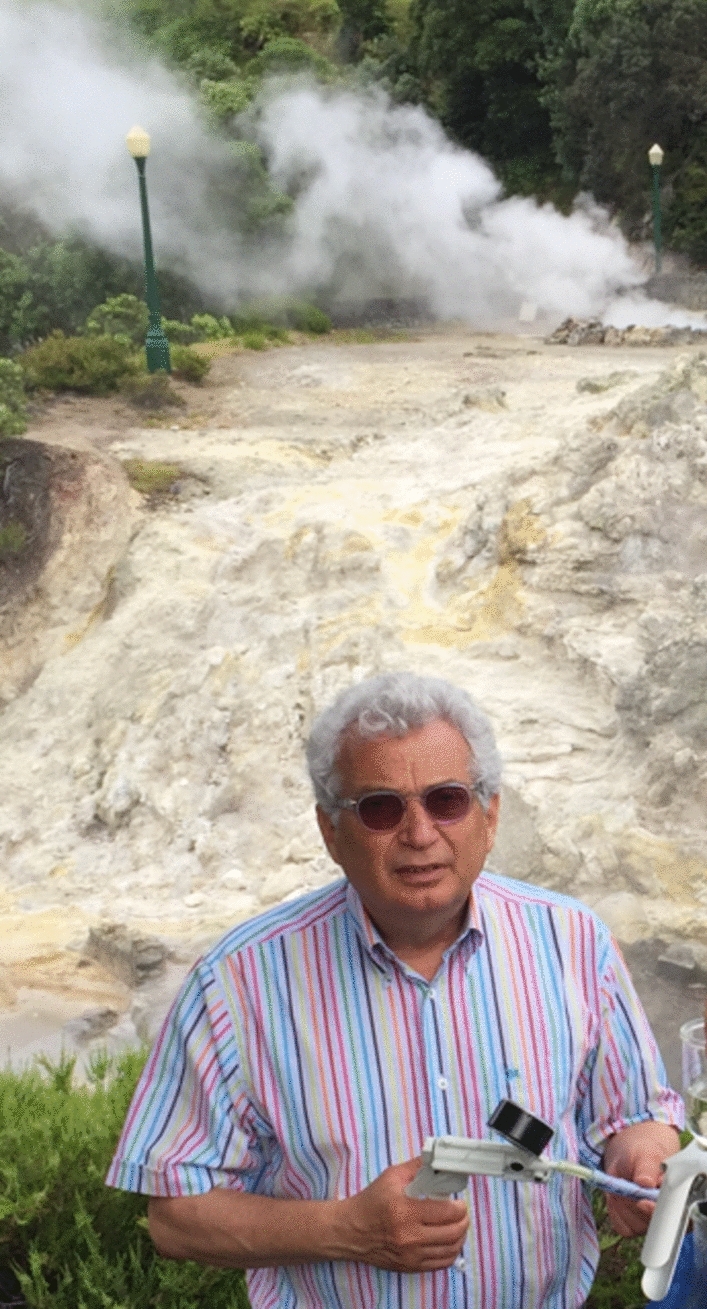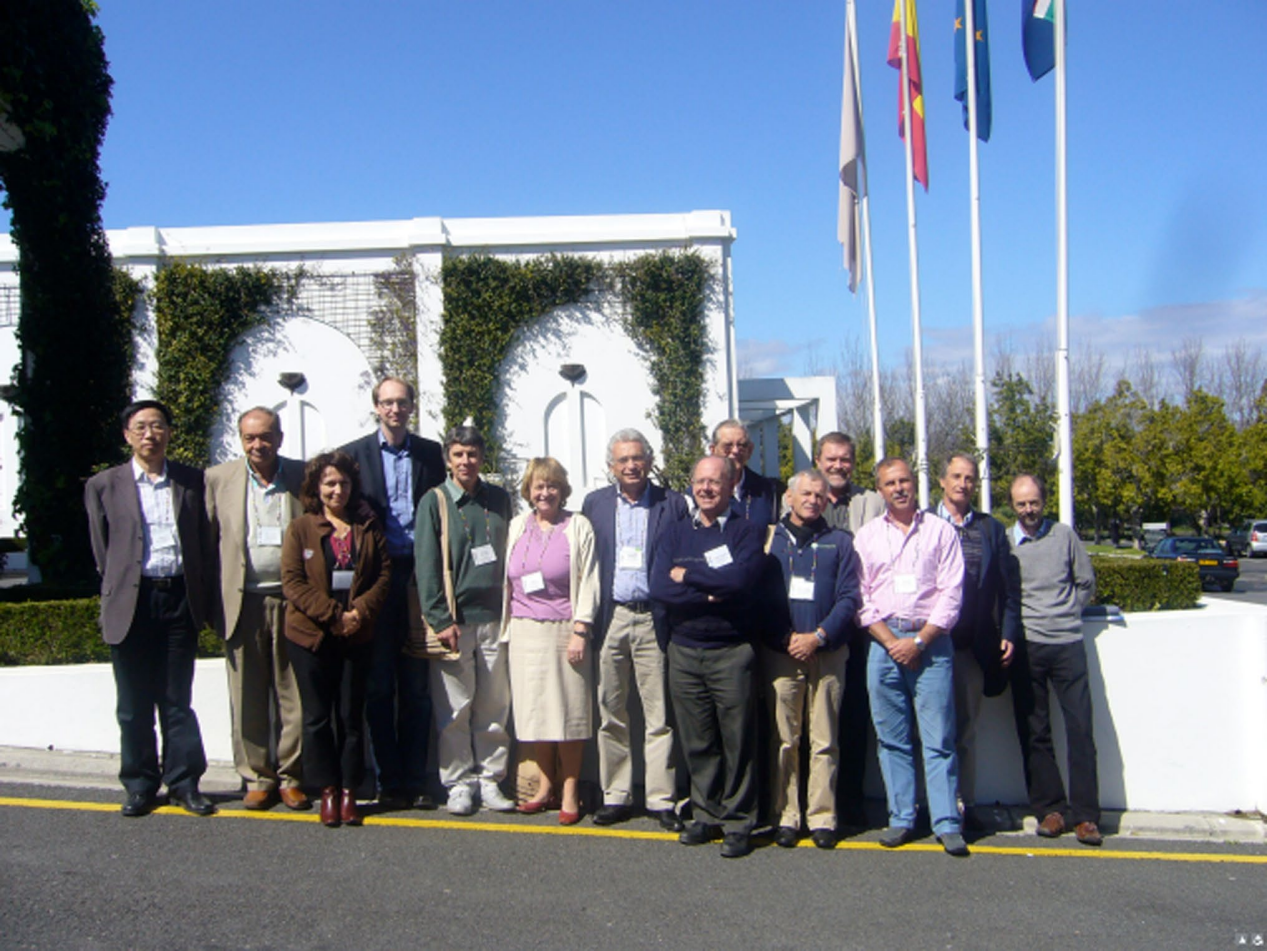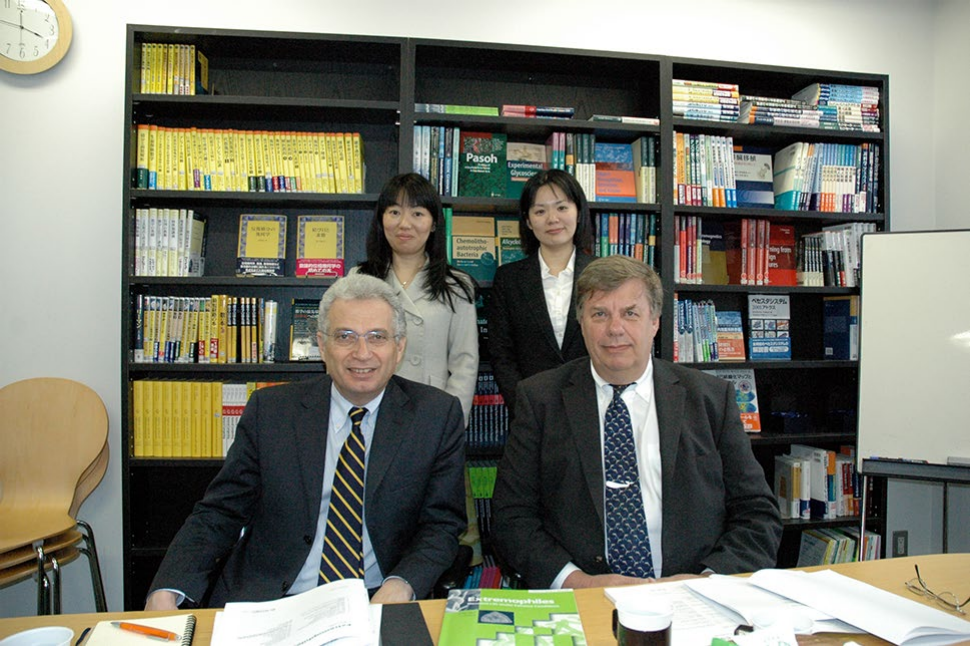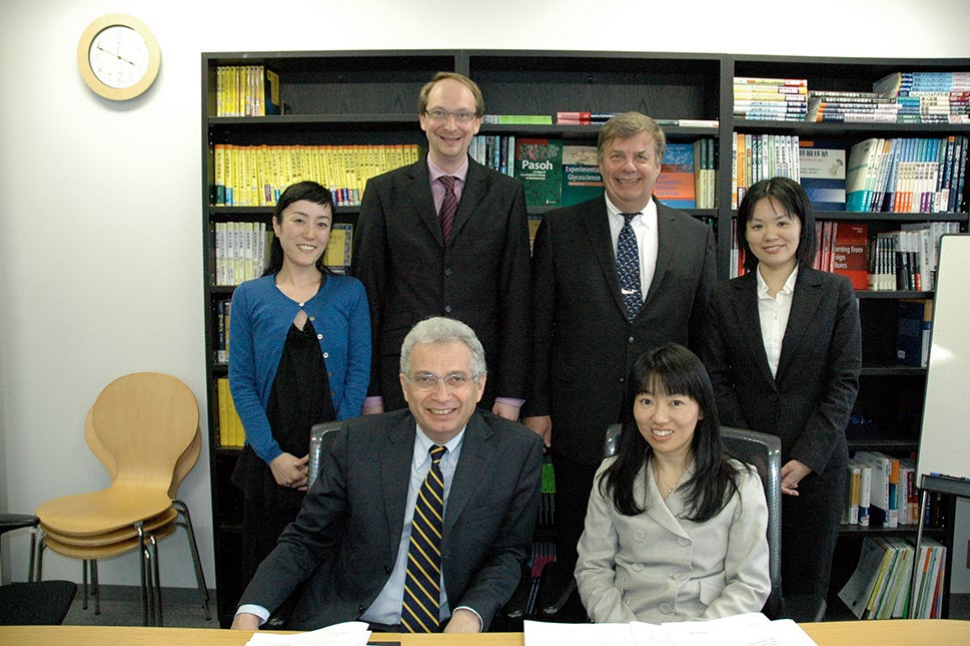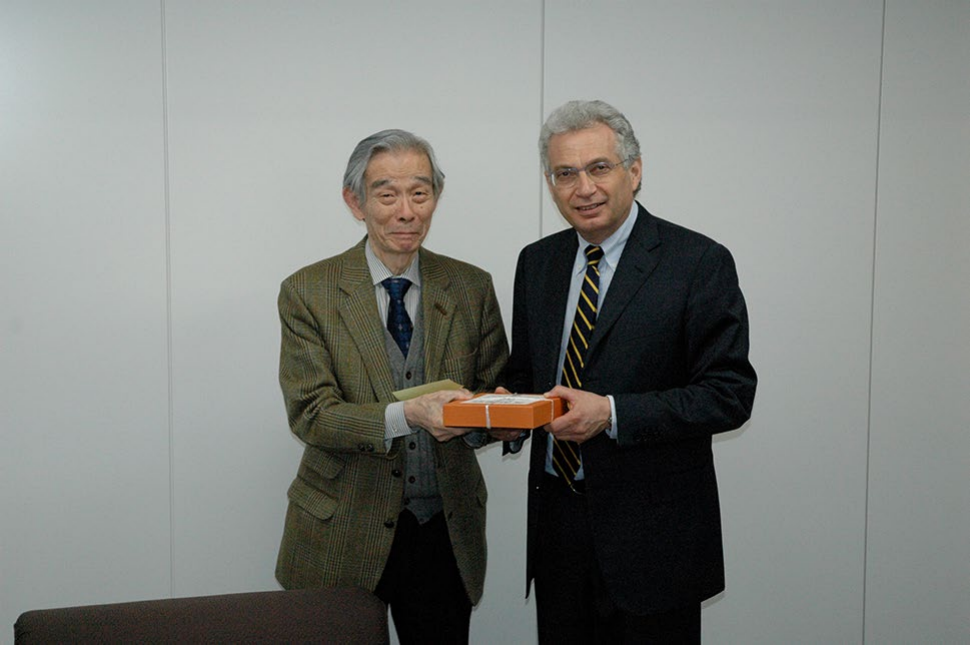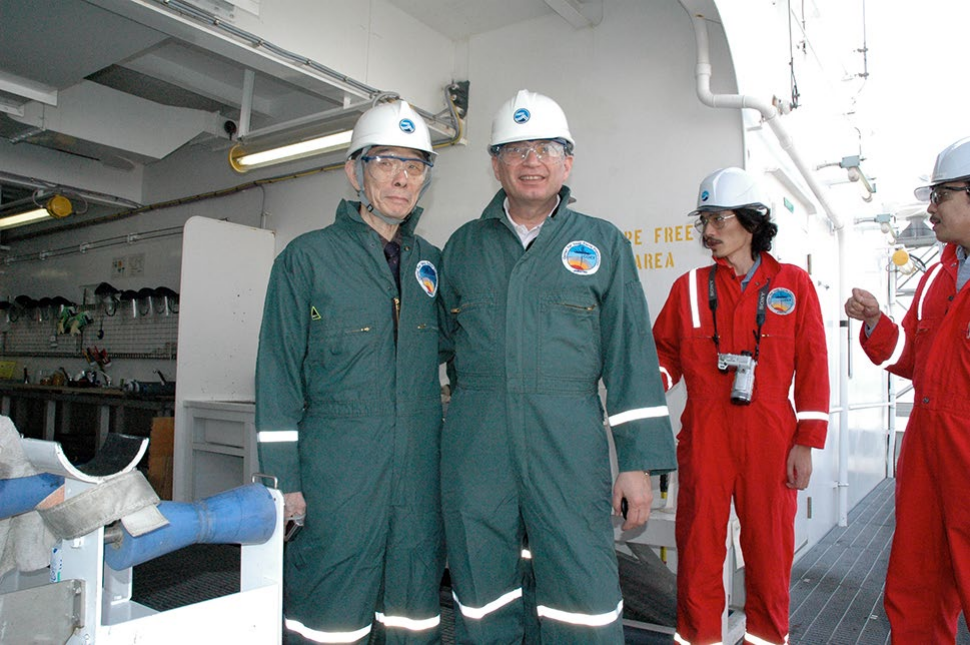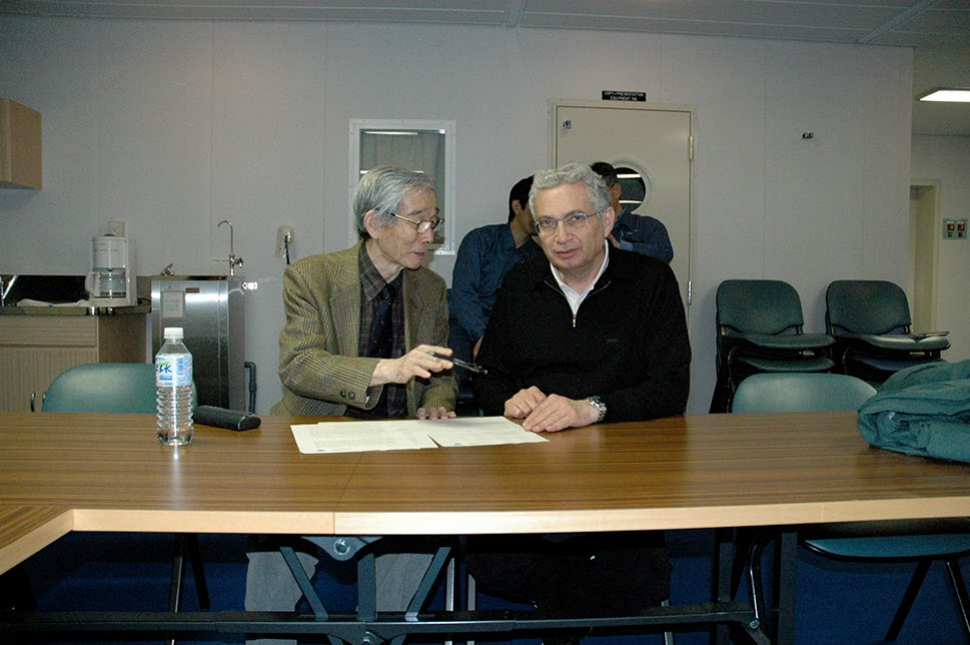# Editorial

**DOI:** 10.1007/s00792-025-01388-0

**Published:** 2025-06-09

**Authors:** Garo Antranikian

**Affiliations:** Technical microbiology, Hamburg University of Technology (TUHH), Hamburg, Germany

Dear Extremophiles Scientific Community,

After more than 17 years of serving as the Chief Editor of Extremophiles Journal, following my esteemed colleague Koki Horikoshi from Japan, in December 2007, I have decided to step down from my role. It has been an incredibly rewarding journey, and I am proud of the success we have achieved together in supporting and advancing research in the field of research on microorganisms thriving under extreme conditions.

Since its launch in 1997, Extremophiles Journal has grown into a leading platform dedicated to research in a fascinating field. The journal has had a significant positive impact on advancing research in both basic and applied microbiology. It has provided the scientific community with cutting-edge information on the latest advancements in the field and has played a crucial role in fostering collaboration and innovation. Furthermore, the journal has helped establish a global network of laboratories and scientists, strengthening the exchange of knowledge and expertise worldwide.

At the initiative of the members of the Editorial Board of the Extremophiles Journal in 2002 „The International Society for Extremophiles (ISE)" was founded. The ISE is intended to be a forum for the exchange of information and experience in the rapidly growing field of research on extremophiles.

This network also laid the foundation for the establishment of the International Conference on Extremophiles, which began in Estoril, Portugal in 1996. Since then, the conference has continued to be held every two years in different locations around the world, serving as a key forum for researchers to share discoveries, develop collaborations, and drive progress in the field. We are looking forward to the upcoming conference which will be in Korea, in September 2026.

I would like to express my heartfelt gratitude to my colleagues and the scientific community for an unwavering support over the years. Your dedication has been instrumental in maintaining the journal’s high scientific standards. It has been a privilege to collaborate with so many of you, and I am especially thankful for your invaluable contributions. It has been a great pleasure to serve this journal from its very first day, and I deeply appreciate the collective efforts that have kept it at the forefront of scientific excellence.

I am truly pleased to announce that Professor Marco Moracci from Naples Italy, will be my successor as Chief Editor. I am deeply grateful for his willingness to take on this responsibility, and I have full confidence that under his leadership, the journal will continue to thrive and evolve.

Wishing you all continued success in the years to come.